# Predicting Disease Severity and Mortality Using a Simple Clinical Risk Score (ABCDMP) in Patients with AECOPD in the UAE

**DOI:** 10.1177/00185787261465850

**Published:** 2026-07-25

**Authors:** Khadeijah Almarshoodi

**Affiliations:** 1 Newcastle University, Sharjah, United Arab Emirates; 2 College of Pharmacy, University of Sharjah, Sharjah, United Arab Emirates

**Keywords:** disease management, respiratory, critical care

## Abstract

Chronic Obstructive Pulmonary Disease (COPD) stands as a leading cause of death globally, with AECOPD significantly worsening patient outcomes. The intersection of COPD with CVDs further escalates the risk, highlighting the need for precise prognostic tools. The ABCDMP score, previously validated in China, offers a simple clinical approach to risk stratification but has not been tested internationally. This study explores the efficacy of the ABCDMP score in predicting disease severity and mortality among AECOPD patients with concurrent CVDs in the UAE. This multicenter retrospective study was conducted in 18 hospitals across the UAE and assessed the predictive accuracy of the ABCDMP score for inpatient mortality, 30-day mortality, and 90-day readmission among AECOPD patients with CVDs. The study included 512 participants, of whom 343 (67.0%) were female, 391 (76.4%) were aged 65 years or older, and 89 (17.4%) had a hospital stay of more than 30 days. The ABCDMP score was evaluated using the Area Under the Receiver Operating Characteristic (AUROC). The ABCDMP score curves for inpatient death, 30-day death, and 90-day readmission were 0.55 (95% CI: 0.49-0.61), 0.48 (95% CI: 0.40-0.55), and 0.58 (95% CI: 0.52-63), respectively. Significant differences in mortality rates were observed among patients with different lengths of stay (*P* = .012) and in abilities to wash independently (*P* = .001), dress (*P* = .001), and feed (*P* = .001). Moreover, the analysis revealed significant differences in the occurrence of patient deaths across various comorbidities, notably renal diseases (*P* = .003) and atrial fibrillation (*P* = .001). The ABCDMP score’s effectiveness in predicting mortality and readmission rates among AECOPD patients with cardiovascular conditions was found to be limited, indicating the need for its enhancement or the development of more accurate tools.

## Introduction

Chronic Obstructive Pulmonary Disease (COPD) is a respiratory illness commonly encompassing emphysema and chronic bronchitis, marked by obstructed airflow leading to breathing difficulties such as shortness of breath and wheezing.^1^ In 2019, COPD ranked as the third leading cause of mortality globally, resulting in approximately 3.23 million deaths.^1^ Acute exacerbation of COPD (AECOPD) is a significant worsening of symptoms, often triggered by infections or environmental pollutants. A 2-year mortality rate of 39.7% was reported among North Indian patients following discharge after AECOPD, indicating high long-term mortality in this population.^2^

In the United Arab Emirates (UAE), it is estimated that the prevalence of COPD ranges from 3.7% to 5.3%.^2^ The condition is particularly prevalent among men, individuals who smoke, and those who have been exposed to gasoline vapors or dust. Additionally, they are a leading cause of mortality, with a significant prevalence of risk factors in the population. A study examining data from the Abu Dhabi Screening Program for Cardiovascular Risk Markers between 2009 and 2015 found a high proportion of modifiable risk factors such as hypertension, smoking, high cholesterol, and obesity among participants.^3^ The study highlighted that a large majority of the population has one or more major cardiovascular risk factors, indicating a higher risk for coronary heart diseases (CHDs) over 10 years compared to international estimates.

Cardiovascular diseases (CVDs) significantly contribute to morbidity and mortality in chronic obstructive pulmonary disease (COPD), with patients facing an increased risk of cardiovascular complications and exacerbations of COPD. This interplay between CVDs and COPD exacerbates patient outcomes, leading to faster disease progression, longer hospital stays, and higher mortality rates. Recognizing the prevalence of CVDs in COPD patients, several prognostic models such as BAP-65,^4^ CURB-65,^5^ DECAF,^6^ and the Noninvasive Ventilation Outcomes (NIVO) score^7^ have been developed to stratify risk and predict in-hospital mortality for AECOPD patients. Nonetheless, these models are designed to forecast in-hospital mortality among all patients with AECOPD, irrespective of their specific conditions. Recently, Zhang et al.^8^ established and tested a new, straightforward clinical score for accurately determining the risk of short-term negative outcomes in patients hospitalized with AECOPD and cardiovascular diseases (CVDs), using data from a forward-looking multicenter cohort study in China. The ABCDMP score had significantly better predictivity for in-hospital mortality than the BAP-65, CURB-65, DECAF, and NIVO scores. However, its effectiveness has been tested only within a single country and solely for predicting mortality. Recognizing the need for broader validation, this study seeks to test the ABCDMP score’s predictive accuracy globally for inpatient mortality, 30-day mortality, and 90-day readmission. This endeavor is crucial for ensuring the score’s robustness and applicability across diverse healthcare settings, ultimately aiming to enhance patient outcomes worldwide through improved risk stratification.

## Methods

### Study Design and Participants

**Figure 1.**
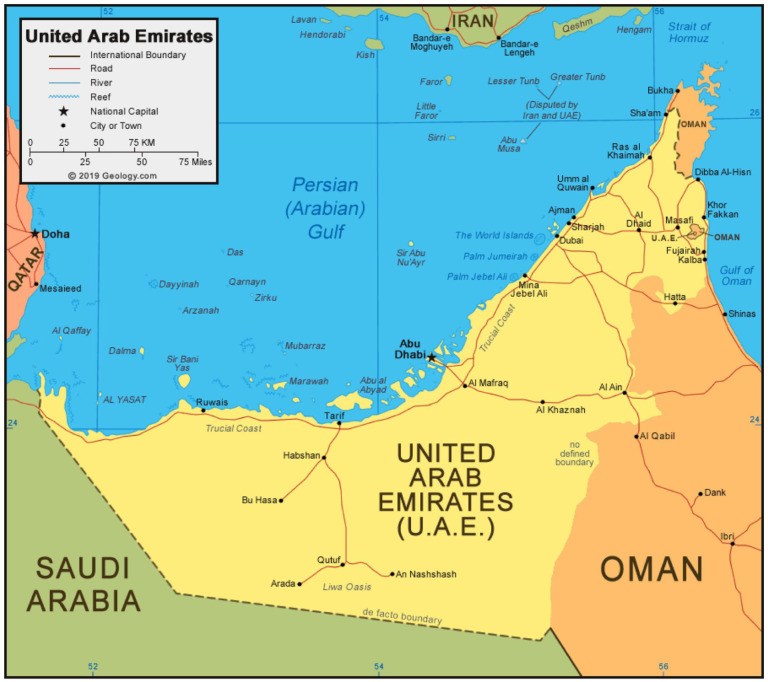
Map of the United Arab Emirates (adapted from Geology.com).^9^ Arrows indicate the cities in which the participating hospitals included in the study were located: Dubai, Umm Al Quwain, Sharjah, Fujairah, and Ras Al Khaimah.

This was a multicenter retrospective observational study conducted across 18 hospitals in the United Arab Emirates (UAE). Data were obtained from the electronic medical records (EMRs) of patients diagnosed with acute exacerbation of chronic obstructive pulmonary disease (AECOPD) and cardiovascular diseases (CVDs) who were admitted to hospitals across six Emirates in the UAE (Figure 1). The participating hospitals included Al-Amal, Al-Kuwait, Al-Qassimi, Al-Dhaid, Khorfakkan, Kalba, Kuwait, Ibrahim Bin Hamad Obaidullah, Abdullah Omran, Obaidalla Geriatric, Shaam, Saqr, Masafi, Dibba, Fujairah, Dubai, Rashid, Latifa Women and Children, and Hatta. These institutions represent secondary and tertiary care hospitals operating within the Emirates Health Services (EHS) and Dubai Health Authority healthcare systems.

All patients received care in accordance with UAE national standards of practice and internationally recognized evidence-based guidelines for AECOPD management, including the recommendations of the Global Initiative for Chronic Obstructive Lung Disease (GOLD), which is widely accepted as the international standard for the diagnosis, management, and prevention of COPD. Pharmacological and supportive treatment decisions were made by the treating physicians in accordance with institutional protocols and current guideline recommendations.

Patients were initially identified through the hospital EMR systems using standardized disease coding for AECOPD and CVD diagnoses, in addition to the EHS AECOPD database. Subsequently, a comprehensive review of medical records was conducted to verify diagnoses and confirm eligibility. The diagnosis of COPD and AECOPD was established by the treating physicians according to GOLD recommendations and standardized institutional clinical practice, based on documented medical history, respiratory symptoms, physical examination findings, laboratory investigations, radiological imaging when indicated, and previous spirometric confirmation of COPD where available.

AECOPD was defined according to GOLD guidelines as an acute worsening of respiratory symptoms beyond normal day-to-day variation that required additional treatment, emergency department evaluation, or hospitalization. To minimize diagnostic misclassification, patients whose respiratory symptoms were attributable solely to alternative diagnoses, such as isolated acute heart failure exacerbation, pneumonia without concurrent AECOPD, or other non-COPD respiratory disorders, were excluded unless AECOPD was clearly documented by the treating physician. AECOPD could be recorded as either the primary reason for admission or a concurrent diagnosis during hospitalization.

The cardiovascular conditions considered included coronary artery disease, heart failure, valvular heart disease, arrhythmias, and stroke, as categorized by the American Heart Association.

Patients with documented terminal or life-limiting illnesses associated with an expected survival of less than 1 year at the time of hospitalization were excluded. Eligibility for this exclusion criterion was determined based on the treating physician’s documented clinical assessment in the medical record. Examples included metastatic or end-stage malignancy receiving palliative care, end-stage organ failure not eligible for definitive treatment (eg, advanced heart, liver, or renal failure), and other terminal conditions with an estimated life expectancy of less than 1 year. This exclusion criterion was applied to reduce potential confounding arising from mortality unrelated to AECOPD or cardiovascular disease and to improve the interpretability of study outcomes and risk prediction analyses.

### Ethics

The study protocol was approved by the federal and local ethics committees: the Research Ethics Committees in the Emirates Health Services (EHS) (EHS/DXB-REC/JAA/No. 29.2019) and the Dubai Health Authority (DHA) (USRRC12-38/PhD/2020).

### Study Outcomes and Data Collection

The primary outcome of the study was the validation of the ABCDMP score for predicting inpatient mortality among patients hospitalized with AECOPD and cardiovascular comorbidities. Secondary outcomes included the evaluation of the ABCDMP score for predicting 30-day mortality and 90-day hospital readmission. In addition, exploratory analyses were conducted to assess the association between selected clinical and laboratory parameters and ABCDMP score categories. These analyses included comparisons of pH levels, eosinophil counts, C-reactive protein (CRP), and urea concentrations across patients with different ABCDMP scores. Demographic and clinical characteristics of the study population were also described, including sex, age, smoking status, mortality outcomes, indicators of disease severity, physical activity capability, body mass index (BMI), vital signs, laboratory parameters (including sodium, potassium, urea, creatinine, albumin, bilirubin, troponin, CRP, hemoglobin, white blood cell count, hematocrit, platelet count, neutrophil count, eosinophil count, pH, PaO_2_, PaCO_2_, and HCO₃), medication use, and comorbid conditions.

ABCDMP scoring criteria encompass six risk factors: Age over 75 years and blood urea nitrogen (BUN) levels above 7 mmol/L, each contributing 1 point; lung consolidation and diastolic blood pressure (DBP) of 60 mmHg or less, each adding 2 points; altered mental status, which adds 3 points; and a pulse rate exceeding 109 beats per minute, which contributes 1 point. The total possible score ranges from 0 to 10 points, with higher scores indicating a greater risk of poor outcomes. In the original validation study, each one-point increase in the ABCDMP score was associated with an 86.3% increase in the odds of mortality (OR 1.863), demonstrating a graded relationship between score magnitude and adverse outcomes.

### Data Management and Analysis

First, the data were imported into a spreadsheet using Microsoft Excel (2018) and subsequently cleaned for accuracy. The data cleaning process included identifying and removing duplicate records, checking for data entry errors and implausible values, verifying consistency between variables, and standardizing variable coding and formats. The cleaned dataset was then transferred into the Statistical Package for the Social Sciences (SPSS), version 26 (IBM Corp., Armonk, NY, USA), for further analysis. Variables with more than 50% missing data were excluded from the analysis. For the remaining variables, missing values accounted for [X%] of the total dataset and were assumed to be missing at random (MAR). To address this missing data, multiple imputation using the Markov Chain Monte Carlo (MCMC) method was performed, as recommended for datasets with arbitrary patterns of missingness and approximately multivariate normal distributions.^10^ The MCMC approach was selected because the proportion of missing data was low to moderate and met the assumptions required for valid imputation. Sensitivity analyses demonstrated no substantial differences between the imputed and complete-case datasets.

The analysis began with a detailed descriptive review, outlining the participants’ demographic and health-related characteristics. This included enumerating absolute counts (n) (alongside their respective percentages (%) for categorical data, while continuous data were summarized as mean values with standard deviations.

Subsequently, an evaluation was conducted to compare the average length of hospital stay (LOS) and clinical laboratory parameters among patients categorized by their ABCDMP scores. The variables assessed included pH, PaO₂, PaCO₂, HCO₃, eosinophil count, neutrophil count, white blood cell count, hemoglobin, hematocrit, platelet count, C-reactive protein (CRP), urea, creatinine, sodium, potassium, albumin, bilirubin, and troponin levels. Patients were grouped by ABCDMP score categories (specify the categories used, e.g., 0-2, 3-5, 6-8, and 9-10, or individual score values if applicable). The variables assessed included pH levels, eosinophil counts, C-reactive protein (CRP), urea concentrations, and other relevant laboratory parameters. One-way analysis of variance (ANOVA) was used to compare the mean values of these variables across the ABCDMP score groups. A *P*-value < .05 was considered statistically significant.

The validation of the ABCDMP score was conducted using the Area Under the Receiver Operating Characteristic (AUROC) curve to assess its ability to discriminate between patients who did and did not experience inpatient mortality, 30-day mortality, and 90-day readmission within the study cohort. While the original ABCDMP model was developed and validated to predict in-hospital and 3-year mortality, the present study additionally evaluated its performance in predicting 30-day mortality and 90-day readmission as exploratory outcomes in an external population of patients with AECOPD and cardiovascular comorbidities. No previously established risk estimates for 30-day mortality or 90-day readmission were available from the original derivation study; therefore, these outcomes were assessed using the observed events recorded in our cohort. The AUROC curve is a robust method for assessing the discriminatory performance of a prediction model by plotting sensitivity against 1-specificity across a range of threshold values. Sensitivity represents the ability of the model to correctly identify patients who experienced the outcome (true positives), whereas specificity reflects its ability to correctly identify patients who did not experience the outcome (true negatives). The AUROC provides a summary measure of model discrimination, with a value of 1.0 indicating perfect discrimination and a value of 0.5 indicating performance no better than chance.

For the analysis, the ABCDMP score was quantified on a scale from 0 to 10 and designated as a categorical variable. The outcomes studied included inpatient mortality, 30-day mortality, and 90-day readmission rates. The Hosmer-Lemeshow goodness-of-fit test was employed to assess model calibration by comparing observed and expected event frequencies across risk strata (deciles) generated from the ABCDMP score. These subgroups were created by ranking patients according to their ABCDMP scores and dividing them into groups of increasing predicted risk. The Hosmer-Lemeshow test evaluates the agreement between predicted and observed outcomes and serves as a measure of calibration. Good calibration indicates close agreement between expected and observed event rates, whereas poor calibration suggests discrepancies that may indicate reduced predictive performance within the study population. For inpatient mortality, calibration was assessed against the ABCDMP score’s original intended application. Since the original ABCDMP derivation study did not provide prediction equations or estimated probabilities for 30-day mortality or 90-day readmission, these outcomes were evaluated as exploratory external validation endpoints. Consequently, analyses for 30-day mortality and 90-day readmission were based on the observed event rates across ABCDMP risk strata within the study cohort rather than on externally derived predicted probabilities.

Sensitivity, or the ability of a model to identify true positives correctly, is evaluated by the ratio of true positives to the sum of true positives and false negatives, with its value ranging from 0 to 1—higher values signal superior model performance. Specificity gauges the accuracy in identifying true negatives, calculated as the ratio of true negatives to the sum of true negatives and false positives, with values also ranging from 0 to 1. Higher specificity values denote a more effective model.

## Results

The study included 512 participants, of whom 343 (67.0%) were female, 391 (76.4%) were aged 65 years or older, and 89 (17.4%) had a hospital stay exceeding 30 days (Table 1). Most patients exhibited impaired exercise tolerance, with 473 participants (92.3%) affected. Exercise tolerance was determined from documented clinical assessments recorded in the electronic medical records by the treating physicians and healthcare providers. Patients were categorized as having impaired exercise tolerance if limitations in performing routine daily activities or physical exertion due to respiratory symptoms were documented during hospitalization. More than half of the patients (265, 51.8%) reported cough symptoms. The most commonly prescribed medication classes were diuretics (30.6%), statins (19.8%), and beta-blockers (15.0%) (Figure 1).

**Table 1.** Patient Characteristics (N = 512).

Parameter	Total	Inpatient death (Yes)	Inpatient death (No)	*P* value
Sex				.476
Female	343 (67.0)	38 (22.5)	131 (77.5)	
Male	169 (33.0)	87 (25.4)	256 (74.6)	
Age (years)				.113
Above 65	391 (76.4)	102 (26.1)	289 (73.9)	
Below 65	121 (23.6)	23 (19.0)	98 (81.0)	
Length of stay (days)				.012
>30	89 (17.4)	31 (34.8)	58 (65.2)	
<30	423 (82.6)	94 (22.2)	329 (77.8)	
Capable of independent washing				.001
Yes	303 (59.2)	94 (31.0)	209 (69.0)	
No	209 (40.8)	31 (14.8)	178 (85.2)	
Capable of independent dressing				.001
Yes	300 (58.6)	94 (31.3)	206 (68.7)	
No	212 (41.4)	31 (14.6)	181 (85.4)	
Capable of independent feeding				.001
Yes	312 (60.9)	99 (31.7)	213 (68.3)	
No	200 (39.1)	26 (13.0)	174 (87.0)	
Exercise tolerance				
Yes	39 (7.7)	18 (46.2)	21 (53.8)	
No	473 (92.3)	107 (22.5)	366 (77.4)	
Confusion				.071
Yes	76 (14.8)	26 (34.2)	50 (65.8)	
No	436 (85.2)	99 (22.7)	337 (77.3)	
Consolidation				.777
Yes	235 (45.9)	56 (23.8)	179 (76.2)	
No	277 (54.1)	69 (24.9)	208 (75.1)	
Cough				.387
Yes	265 (51.8)	71 (26.8)	194 (73.2)	
No	247 (48.2)	54 (21.9)	193 (78.1)	
Diagnosed with diabetes				.060
Yes	233 (45.5)	66 (28.3)	167 (71.7)	
No	279 (54.5)	59 (21.1)	220 (78.9)	
Diagnosed with renal disease				.003
Yes	140 (27.3)	47 (33.6)	93 (66.4)	
No	372 (72.7)	78 (21.0)	294 (79.0)	
Diagnosed with heart failure				
Yes	72 (14.1)	24 (33.3)	48 (66.7)	
No	440 (85.9)	101 (23.0)	339 (77.0)	
Diagnosed with atrial fibrillation				.001
Yes	232 (45.3)	96 (41.4)	136 (58.6)	
No	280 (54.7)	29 (10.4)	251 (89.6)	

Abbreviations: ADL, activities of daily living; AECOPD, acute exacerbation of chronic obstructive pulmonary disease.

Values are presented as n (%). *P*-values were calculated using the Chi-square test for categorical variables. Exercise tolerance was determined from documented clinical assessments recorded in the electronic medical records by treating physicians and healthcare providers.

The ABCDMP score demonstrated limited discriminatory performance for predicting inpatient mortality, 30-day mortality, and 90-day readmission among patients with AECOPD and cardiovascular comorbidities. Comparisons between patients who died and those who survived revealed significant differences in several clinical characteristics. Specifically, significant differences were observed in length of hospital stay (*P* = .012) and in functional status measures, including independent washing (*P* = .001), dressing (*P* = .001), and feeding (*P* = .001). Furthermore, significant differences in mortality rates were identified among patients with specific comorbidities, particularly renal disease (*P* = .003) and atrial fibrillation (*P* = .001). These comparisons were performed using Chi-square tests for categorical variables and independent-samples *t*-tests or Mann-Whitney *U* tests for continuous variables, as appropriate.

**Figure 2.**
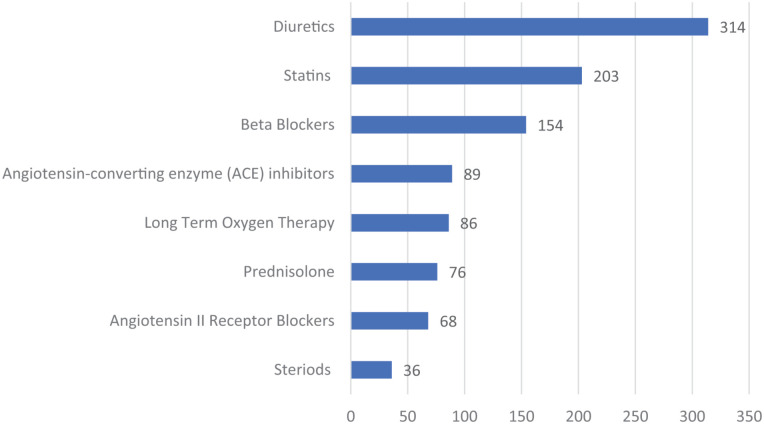
List of drugs prescribed for patients included in the study (N = 1026) (each patient may have one or more medications). Abbreviations: N, total number of prescribed medications. Patients may have received more than one medication; therefore, the total number of prescriptions exceeds the total number of patients.

The ABCDMP score curves for inpatient death, 30-day death, and 90-day readmission were 0.55 (95% CI: 0.49-0.61), 0.48 (95% CI: 0.40-0.55), and 0.58 (95% CI: 0.52-63), respectively (Table 2 and Figure 2). The model demonstrated poor calibration in the study cohort, as indicated by the Hosmer-Lemeshow goodness-of-fit statistic (χ^2^ = 20.5) and a Nagelkerke *R*^2^ value of .15 (Table 2). These findings suggest limited agreement between the observed and expected outcomes predicted by the ABCDMP score in this population. However, the Hosmer-Lemeshow test should be interpreted with caution, as calibration assessments may be influenced by sample size and event frequency. Compared with the original prospective ABCDMP derivation study, which included several thousand patients, the present external validation cohort was substantially smaller (n = 512), potentially limiting the stability of calibration estimates. As shown in Table 2, increasing ABCDMP scores were not consistently associated with higher rates of in-hospital mortality, 30-day mortality, or 90-day readmission. For example, the highest in-hospital mortality rate was observed among patients with an ABCDMP score of 2 (5.3%), rather than among those with the highest score categories.

**Table 2.** Survival Rate During Hospitalisation According to the ABCDMP score.

ABCDMP score	Total, n (%)	In patient death	30-day death	90-day readmission
0	17 (3.3)	5 (1.0)	5 (1.0)	6 (1.2)
1	88 (17.2)	16 (3.1)	14 (2.7)	24 (4.7)
2	103 (20.1)	27 (5.3)	22 (4.3)	37 (7.2)
3	97 (18.9)	19 (3.7)	24 (4.7)	31 (6.1)
4	99 (19.3)	21 (4.1)	20 (3.9)	40 (7.8)
5	43 (8.4)	13 (2.5)	10 (1.9)	17 (3.3)
6	29 (5.7)	9 (1.8)	9 (1.8)	16 (3.1)
7	22 (4.3)	7 (1.4)	6 (1.2)	9 (1.8)
8	8 (1.6)	5 (1.0)	5 (1.0)	4 (0.8)
9	4 (0.8)	3 (0.6)	2 (0.4)	2 (0.4)
10	2 (0.4)	0 (0.0)	0 (0.0)	1 (0.2)
*Risk category*				
Low (0-1)	105 (20.5)	21 (4.1)	19 (16.2)	27 (5.3)
Medium (2-4)	299 (58.4)	67 (13.1)	66 (28.1)	108 (21.1)
High (>4)	108 (21.1)	37 (7.2)	32 (13.6)	49 (9.6)
AUC (95% CI)	—	0.556 (0.495-0.616)	0.481 (0.407-0.555)	0.580 (0.529-0.632)

Abbreviations: ABCDMP, age, blood tests, comorbidities, dyspnea, mental status, and performance status score; AUC, area under the receiver operating characteristic curve; CI, confidence interval.

Values are presented as n (%). Low-risk category = ABCDMP score 0-1; medium-risk category = ABCDMP score 2-4; high-risk category = ABCDMP score >4.

Table 3 presents comparisons of clinical and laboratory parameters across ABCDMP score categories using one-way analysis of variance (ANOVA). No consistent trend was observed between increasing ABCDMP scores and admission pulse rate, temperature, body mass index (BMI), oxygen saturation (SpO₂), serum creatinine, albumin, hematocrit, hemoglobin, C-reactive protein (CRP), troponin, or eosinophil counts. However, statistically significant differences were identified across ABCDMP score groups for admission respiratory rate (*P* = .012), blood urea nitrogen (BUN) (*P* = .001), and fasting glucose levels (*P* = .003). Patients with an ABCDMP score of 10 demonstrated the highest mean respiratory rate (32 breaths/min) and pulse rate (114 beats/min). Detailed group-specific means, standard deviations, and corresponding *P*-values are presented in Table 3.

**Table 3.** Laboratory Markers Versus ABCDMP Score.

ABCDMP score	Admission pulse rate (bpm)	Admission respiratory Rate	Admission temperature (℃)	BMI	SpO₂	BUN	Cr	Albumin	Glucose fasting at admission	Hct	Hb	CRP	Troponin	Eosinophil
0	91.06 (12.002)	21.06 (4.205)	37.07 (0.666)	29.69 (8.206)	92.5 (3.0)	4.14 (1.8805)	222.17 (203.22)	31.84 (11.19)	11.58 (21.87)	38.43 (6.22)	14.32 (8.62)	50.32 (48.83)	476.58 (753.76)	0.41 (0.552)
1	98.84 (25.864)	23.08 (6.463)	36.87 (0.487)	31.60 (13.25)	90.14 (8.055)	6.97 (4.1828)	134.68 (110.35)	34.96 (29.07)	26.90 (42.40)	37.78 (7.76)	14.28 (19.43)	73.66 (75.38)	783.47 (2559.49)	0.29 (0.721)
2	110.38 (34.002)	23.17 (6.735)	37.01 (0.591)	30.59 (11.90)	88.84 (13.28)	7.56 (3.9088)	185.20 (404.53)	34.28 (29.02)	31.94 (56.32)	37.31 (7.18)	13.06 (8.60)	71.93 (85.48)	659.26 (1606.38)	0.18 (0.220)
3	106.82 (35.491)	23.72 (7.482)	37.04 (0.713)	29.88 (8.56)	90.03 (9.212)	8.23 (5.1081)	145.31 (143.19)	30.08 (8.60)	40.77 (74.58)	36.36 (7.90)	12.25 (4.54)	71.78 (87.93)	624.69 (1305.91)	0.17 (0.192)
4	111.83 (35.459)	23.83 (6.331)	36.92 (0.552)	32.86 (22.35)	87.85 (10.32)	9.15 (4.2389)	184.55 (527.82)	30.54 (12.63)	51.29 (73.89)	38.20 (20.18)	11.90 (2.64)	75.22 (88.57)	570.36 (1287.27)	0.26 (0.407)
5	107.42 (34.723)	22.56 (8.075)	37.02 (0.643)	29.45 (7.30)	92.7 (5.794)	10.22 (7.1299)	137.38 (81.13)	31.66 (8.88)	32.95 (49.53)	37.90 (7.48)	12.38 (3.43)	83.28 (90.93)	594.59 (949.90)	0.17 (0.230)
6	100.17 (25.499)	23.69 (6.234)	36.94 (0.623)	30.07 (8.84)	85.38 (13.05)	11.81 (9.6824)	208.28 (203.63)	29.15 (10.11)	32.71 (55.01)	36.45 (6.11)	11.60 (2.13)	98.89 (88.35)	470.38 (656.51)	0.18 (0.252)
7	110.27 (27.461)	22.00 (2.992)	37.11 (0.778)	26.70 (10.59)	86.67 (9.209)	14.01 (12.8967)	106.79 (62.40)	31.81 (9.56)	50.11 (76.27)	34.79 (4.87)	11.24 (1.69)	70.97 (75.11)	731.93 (1182.03)	0.22 (0.247)
8	100.75 (30.245)	24.12 (6.198)	37.15 (1.384)	28.93 (6.80)	81.33 (11.72)	11.68 (11.3434)	260.11 (189.73)	25.42 (7.14)	33.98 (46.34)	37.90 (9.19)	10.22 (4.09)	103.43 (87.21)	684.38 (1325.44)	1.44 (3.106)
9	106.00 (27.289)	20.00 (2.708)	37.98 (1.201)	34.18 (9.96)	94.33 (8.963)	20.95 (29.7982)	129.44 (15.64)	29.10 (8.94)	66.27 (64.18)	29.97 (6.07)	10.88 (1.45)	36.70 (46.07)	173.45 (203.56)	0.24 (0.375)
10	114.00 (2.828)	32.00 (0.0)	36.10 (0.566)	25.50 (10.89)	91.00 (N/A)	12.45 (6.4347)	53.05 (12.51)	32.00 (2.83)	142.00 (67.88)	38.30 (1.13)	11.55 (0.354)	36.40 (23.48)	22.00 (8.49)	0.30 (0.283)

## Discussion

The link between COPD and CVDs is well documented and represents a clinically important interaction that adversely affects patient outcomes. Both conditions share several risk factors, including smoking, systemic inflammation, sedentary lifestyle, aging, and oxidative stress. These common etiological pathways contribute to increased cardiovascular mortality and a higher risk of acute cardiovascular events during COPD exacerbations. Consequently, a multidisciplinary approach to diagnosis and management is essential for optimizing patient outcomes.

In this study, we evaluated the performance of the ABCDMP score in predicting inpatient mortality, 30-day mortality, and 90-day readmission among patients hospitalized with AECOPD and coexisting cardiovascular diseases. To our knowledge, this is among the first external validation studies assessing a prognostic tool specifically developed for patients with both AECOPD and cardiovascular comorbidities.

**Figure 3.**
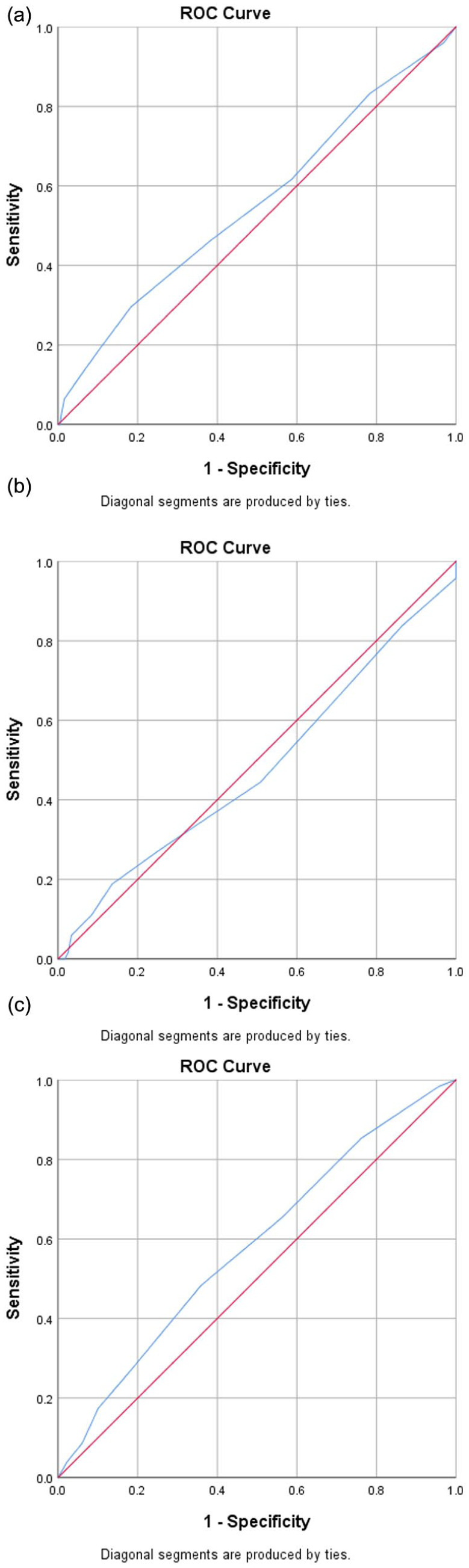
Receiver operating characteristic (ROC) curves for the ABCDMP score in predicting inpatient mortality, 30-day mortality, and 90-day readmission. The ROC curves illustrate the discriminatory performance of the ABCDMP score for each outcome. Curves were generated by plotting sensitivity (true positive rate) against 1 − specificity (false positive rate) across a range of threshold values. The area under the ROC curve (AUROC) was used to assess the predictive accuracy of the ABCDMP score for each outcome: (a) ROC curve for mortality, (b) 30-day mortality ROC curve, and (c) 90-day readmission ROC curve. Abbreviations: ABCDMP, age, blood tests, comorbidities, dyspnea, mental status, and performance status score; AUROC, area under the receiver operating characteristic curve; ROC, receiver operating characteristic.

Our analysis demonstrated limited discriminatory performance of the ABCDMP score for predicting inpatient mortality, 30-day mortality, and 90-day readmission. Specifically, the AUROC values of 0.556, 0.481, and 0.580, respectively, indicate poor discrimination, with performance only marginally better than chance for inpatient mortality and 90-day readmission and no meaningful discriminatory ability for 30-day mortality. Furthermore, calibration analyses suggested suboptimal agreement between observed and expected outcomes. These findings indicate that the ABCDMP score may have limited utility for risk stratification in this patient population and suggest that refinement of the existing model or development of alternative prognostic tools may be warranted (Figure 3).

Our findings differ substantially from those reported by Zhang et al.,^8^ who demonstrated excellent discrimination of the ABCDMP score for inpatient mortality (AUROC = 0.847). Several factors may explain this discrepancy. First, differences in patient characteristics, disease severity, healthcare delivery systems, and comorbidity profiles between the derivation and validation cohorts may have influenced model performance. Second, the original ABCDMP model was developed and internally validated in a large cohort comprising several thousand patients, whereas our external validation cohort was substantially smaller. The lower number of outcome events in our study may have reduced the precision and stability of both discrimination and calibration estimates, particularly for subgroup analyses and Hosmer-Lemeshow testing. Third, differences in population demographics, geographic settings, clinical practices, and data availability may have contributed to reduced model transportability. These findings highlight the challenges associated with external validation of prognostic models and emphasize the importance of validating prediction tools across diverse healthcare settings before widespread clinical implementation.

Importantly, although our study demonstrated lower predictive performance than the original derivation study, this does not necessarily invalidate the ABCDMP score. Rather, it suggests that the model’s performance may vary across populations and healthcare systems. Additional multicenter prospective studies with larger sample sizes are needed to further evaluate the generalizability of the score and determine whether recalibration or regional adaptation may improve its predictive accuracy.

Our findings also revealed significant associations between mortality and several markers of patient frailty and disease burden. Patients who died during hospitalization were more likely to have prolonged hospital stays and impaired functional status, as reflected by reduced ability to perform independent washing, dressing, and feeding. These findings are consistent with previous literature indicating that poor functional status and comorbidity burden are associated with worse outcomes in patients with COPD and acute exacerbations.^11^

In addition, significant differences in mortality were observed among patients with renal disease and atrial fibrillation (AF). AF is associated with increased risks of stroke, heart failure, systemic thromboembolism, and overall mortality, all of which may contribute to poorer outcomes during AECOPD admissions.^12^ Similarly, chronic kidney disease has been consistently associated with increased cardiovascular risk, systemic inflammation, and mortality in COPD populations. A systematic review reported a higher prevalence of chronic kidney disease among COPD patients and demonstrated that the coexistence of these conditions substantially increases mortality risk.^13^ The observed associations in our study further support the importance of comprehensive assessment and management of comorbidities when evaluating prognosis in patients with AECOPD and cardiovascular disease.

## Limitations

This study has several limitations. First, we were unable to compare the ABCDMP score with other established prognostic tools using the same dataset. Such comparisons may have provided additional insight into the relative predictive performance of the ABCDMP score in patients with AECOPD and cardiovascular comorbidities.

Second, while this study encompasses a substantial number of hospitals across Dubai and the Northern Emirates, it primarily includes patients treated within the Emirates Health Services and Dubai Health Authority healthcare systems. The exclusion of patients from hospitals in Abu Dhabi due to challenges in obtaining ethical approval may limit the generalizability of the findings to the entire UAE population.

Third, the retrospective design provides valuable insight into real-world clinical practice but is inherently subject to limitations related to data completeness, documentation variability, and potential residual confounding. Although efforts were made to address missing data through multiple imputation using the Markov Chain Monte Carlo method, the potential influence of missing data on study findings cannot be completely excluded.^10,14^

Finally, the external validation cohort was substantially smaller than the original ABCDMP derivation cohort, which included several thousand patients. The relatively modest sample size and lower number of outcome events in the present study may have reduced the precision and stability of discrimination and calibration estimates, particularly those derived from the Hosmer-Lemeshow goodness-of-fit test, which can be sensitive to sample size and event frequency. Consequently, the calibration results should be interpreted with caution. Larger prospective multicenter studies involving broader patient populations are warranted to further evaluate the performance, calibration, and generalizability of the ABCDMP score in patients with AECOPD and cardiovascular comorbidities.

## Conclusion

The ABCDMP score’s predictive power for inpatient mortality, 30-day mortality, and 90-day readmission in AECOPD patients with CVDs was limited, suggesting a need for further refinement or development of more precise predictive tools. Significant mortality rate differences were observed among patients with varying lengths of stay, abilities in activities of daily living, and certain comorbidities, such as renal diseases and atrial fibrillation, underscoring the complex interplay of factors influencing outcomes in this patient population.
